# Cognitive Models in Intelligence Research: Advantages and Recommendations for Their Application

**DOI:** 10.3390/jintelligence6030034

**Published:** 2018-07-17

**Authors:** Gidon T. Frischkorn, Anna-Lena Schubert

**Affiliations:** Institute of Psychology, Heidelberg University, Hauptstrasse 47-51, D-69117 Heidelberg, Germany; anna-lena.schubert@psychologie.uni-heidelberg.de

**Keywords:** intelligence, cognitive modeling, methods, measurement, practical guidelines

## Abstract

Mathematical models of cognition measure individual differences in cognitive processes, such as processing speed, working memory capacity, and executive functions, that may underlie general intelligence. As such, cognitive models allow identifying associations between specific cognitive processes and tracking the effect of experimental interventions aimed at the enhancement of intelligence on mediating process parameters. Moreover, cognitive models provide an explicit theoretical formalization of theories regarding specific cognitive processes that may help in overcoming ambiguities in the interpretation of fuzzy verbal theories. In this paper, we give an overview of the advantages of cognitive modeling in intelligence research and present models in the domains of processing speed, working memory, and selective attention that may be of particular interest for intelligence research. Moreover, we provide guidelines for the application of cognitive models in intelligence research, including data collection, the evaluation of model fit, and statistical analyses.

## 1. Introduction

One of the greatest challenges in intelligence research is the identification of cognitive processes underlying cognitive abilities and the measurement of process parameters giving rise to individual differences in general intelligence [1]. Traditional as well as current theories of general intelligence either assume that intelligent behavior is the result of individual differences in various independent cognitive abilities [2,3,4], or that there is a hierarchical structure of cognitive abilities with a domain general and broad factor of general intelligence *g* that determines individual differences in cognitive abilities [5,6,7,8,9]. Theoretically and empirically the most discussed process parameters related to individual differences in general intelligence are the speed of information processing e.g., [9,10], the capacity of short-term memory e.g., [11], working memory e.g., [12,13,14] or secondary memory e.g., [15,16], and the efficiency of executive functions e.g., [4,17,18].

With respect to these theoretical and empirical considerations, there are three main goals to this process-oriented approach to intelligence research: First, understanding whether *one* or *several* cognitive processes give rise to individual differences in general intelligence will help to decide whether *g* should be conceived of as a single cognitive process, as suggested by Spearman’s two-factor theory [5], or as an emerging phenomenon due to several independent or interacting cognitive processes, as suggested by sampling theories [4,19]. Second, a process-oriented approach aims to identify the mechanisms limiting or facilitating performance in certain cognitive processes by developing formal theories of the mechanisms constituting these processes. Third, such a process-oriented approach may ultimately lead to the development of formal theories of general intelligence by combining psychometric approaches and previous insights into the mechanisms of cognitive processes strongly related to general intelligence.

In empirical research, individual differences in these cognitive processes are usually measured by behavioral indicators such as response times and accuracies in tasks supposedly engaging one specific cognitive process. The behavioral performance in these tasks is then used to quantify the relationship of these cognitive processes to overall performance in intelligence tests. This approach presumes that a specific task provides a process-pure measure of a single cognitive process—an assumption that is often violated as most cognitive tasks do not measure one specific cognitive process, but rather a combination of several cognitive processes. For example, tasks measuring the efficiency of inhibitory processes such as the Stroop or Flanker task usually use reaction times as performance measures [20,21]. These reaction times arguably reflect not only the efficiency of inhibitory processes, but also basic information-processing speed. Another example is complex cognitive tasks such as complex span tasks measuring working memory capacity that not only require the storage of information in the face of processing, but may also rely on attentional control processes and speed of information processing [22,23]. In sum, typical measures for a specific cognitive process thus require additional cognitive processes beyond the cognitive process aimed to be measured.

Two approaches are typically pursued to overcome this problem. First, variance decomposition methods may be used to isolate the variance of one latent cognitive process parameter from the influence of other variables e.g., [11,12,17]. This method is feasible as long as there are “pure” measurements of the confounding cognitive processes available that can be controlled for. However, this approach may be resource- and time-consuming, as participants have to complete large test batteries including both measures of interest and of possible confounds.

A second approach to this measurement problem is to design experimental tasks that contain a baseline condition requiring the engagement of all confounding processes and an experimental condition that is equal to the baseline condition except for the insertion of one additional processing requirement of interest. Subtracting performance in the baseline condition from performance from the experimental condition is supposed to isolate the efficiency or speed of the added process [24]. However, it is questionable if the resulting difference scores only contain variance that can be attributed to the inserted process or if the insertion of additional processing demands may affect or interact with other task demands that are also reflected in the difference scores [25,26]. Moreover, the low between-subject variability and low reliability of difference scores in typical cognitive tasks renders the isolation of individual differences in experimental effects by means of difference scores virtually impossible [27,28].

In the present paper, we aim to demonstrate how mathematical models of cognition can be used to partially overcome these measurement problems by directly quantifying specific cognitive processes. Moreover, we will provide practical guidelines and recommendations for the use of cognitive models in intelligence research. While ultimately a formalization of specific theories of intelligence e.g., [3,4] would be desirable, these theories are still too general and abstractly formulated to allow the development of a formalized cognitive model of intelligence. As long as this is the case, the incorporation of mathematical models of the cognitive processes addressed in these theories provides a first necessary step towards a concrete formalized theory of intelligence. Therefore, the present manuscripts focuses on mathematical models of cognitive processes that are related to general intelligence or *g* rather than on cognitive models for general intelligence itself.

## 2. Advantages of Cognitive Modeling in Intelligence Research

### 2.1. Statistical Models

Although often not explicitly in mind, each measurement of a cognitive process and more generally any property of a person is based on a model. Most often we use statistical models, such as classical test theory or latent variable models for this measurement procedure [29]. These models typically assume that the measured and observed behavior is the compound of some *true* or *latent* property of a person and of an *error* of measurement [30,31,32]. Across repeated measurements of the same property, this results in a distribution of observations of which the average or expected value given a person (i.e., the arithmetic mean) is conceptualized as the best estimate of the *true* person property, while deviations from this value (i.e., the standard deviation) correspond to the amount of *error* or uncertainty in the measurement. Taken together, statistical models describe statistical properties of observed variables such as their mean and reliability (according to classical test theory), or the covariances among different variables (according to latent variable models).

Even though statistical models have proven to be very useful in the context of measurement, such models bear serious conceptual problems [29,33] and the selection of an adequate statistical model for measurement is anything but trivial. Apart from these general philosophical and epistemological problems of measurement with statistical models such as the ontological status of true-scores or latent variables and the adoption of a realist or constructionist perspective on science and measurement [29], all of these models have another serious shortcoming: Statistical models do not specify any psychological or cognitive processes underlying the *true* part of the measurement, but rather focus on separating *true* properties of a person from the *error* of measurement.

In response to this problem, it has been recommended to use more elaborate statistical models such as ex-Gaussian- or Wald- distributions for reaction times [34,35,36], and Binomial-distributions for accuracies or mental test scores [37,38]. Although these distributions correspond more closely to the empirical shape of the distributions of observed variables, the parameters of these distributions do not consistently resemble indicators of distinct cognitive processes, see [39]. More importantly, these models still only describe statistical characteristics of the observed variables and do not provide a theoretical account of the cognitive processes underlying the observed behavior. In sum, statistical models may be useful to quantify the amount of variance in a measurement attributable to the *true* personality trait (i.e., the reliability), however they do not allow any theoretically founded statements about the cognitive processes underlying the observed behavior or the latent personality trait.

### 2.2. Cognitive Models

Conversely, cognitive models may provide a mathematically-guided quantification of specific cognitive processes [40]. Specifically, cognitive models translate explicit verbal theories of cognitive processes in specific tasks into mathematical formulations of these theories. In this, the behavioral measures within a task are described as the result of different interacting processes or parameters of the model. The detailed interplay and interaction of these processes is specified within the formal architecture of the model and represents the assumptions the model makes with respect to a specific cognitive process. Thus, a cognitive model represents a formalized theory of a cognitive process that objectively states which parameters of the cognitive process affect differences in observed behavior across conditions or individuals. The adequacy and validity of this formalization can be evaluated by parameter recovery studies and by testing the selective effects of theoretically-guided experimental manipulations on model parameters [41].

Taken together, cognitive models provide several advantages over statistical models: (1) They provide falsifiable descriptions of the cognitive process underlying behavioral responses in a specific task; (2) Model parameters can be interpreted in an objective and formally described manner; and (3) Model parameters can be used to quantify individual differences in specific cognitive processes based on the underlying model architecture.

## 3. Selecting Cognitive Models Suitable for Intelligence Research

Usually, cognitive models are used with two different aims: (1) A cognitive model aims to *formally describe* the cognitive processes underlying the observed behavior in a specific task and *explain* specific experimental effects observed within this task; (2) The parameters of a cognitive model estimated from the observed behavior in a task are used as *measures* for differences across individuals or experimental conditions. These measures quantify how far people or conditions differ with respect to a specific process of the cognitive model. Within the field of cognitive modeling, cognitive models serving the first aim are often described as explanatory cognitive models or cognitive process models, while cognitive models used with the second aim are often called cognitive measurement models [42]. Accordingly, any cognitive model can be considered both an explanatory cognitive model and a cognitive measurement model depending on the circumstances of its use. Nevertheless, cognitive models that are used to explain the observed behavior within a specific task (i.e., explanatory cognitive models) often differ from cognitive models that are used to quantify differences in their parameters across individuals or conditions (i.e., cognitive measurement models).

In detail, explanatory cognitive models aim to provide formal explanations for variations across experimental conditions in specific paradigms in terms of cognitive processes. These models formally describe the architecture of a cognitive process and focus on the interplay of different mechanisms that lead to specific experimental results. In contrast, cognitive measurement models typically decompose the observed behavior of a person into meaningful parameters of a latent cognitive process. Thus, instead of explaining differences across individuals or experimental conditions, cognitive measurement models are highly flexible tools that reflect these differences in variations of their estimated parameters (for a comparison of these two model types, see [43]). Often cognitive measurement models rely on a more elaborated explanatory cognitive model. However, there are many cognitive measurement models that have been developed independently of any explanatory cognitive model e.g., [44].

With respect to their application, cognitive models used to explain observed behavior, such as the SOB-CS [45], the slot-averaging model [43], or the interference model of visual working memory [42], often resemble highly elaborated model architectures that specify detailed formal models for a cognitive process. These models are often very complex and require high computing power to calculate predictions for a given set of parameters. In contrast, cognitive models used to measure differences of parameters across individuals or conditions, such as signal-detection theory [44], the two-high threshold model for recognition [46], or the drift-diffusion model [47], are mostly simplified descriptions of a cognitive process that can be generalized to a broad set of paradigms and observed variables. Beyond that, such cognitive measurement models are easy to use and parameters of cognitive measurement models can either be readily calculated from observed variables or estimated with adequate fitting procedures.

In intelligence research, the use of cognitive measurement models is far more widespread than the use of explanatory cognitive models. Although explanatory cognitive models provide a powerful tool for comparing different theories with respect to their predictions for experimental paradigms and manipulations, see [48]; their complexity and especially the lack of estimable parameters renders their application in intelligence research difficult. Still, results from explanatory cognitive models may provide the theoretical foundation for deciding for or against a specific cognitive measurement model.

Furthermore, there have been efforts to formulate explanatory models of intelligence test performance such as the Carpenter et al. [49] model for performance in the Raven matrices. In this model, Carpenter et al. [49] described different cognitive processes that are used while solving the Raven matrices. Some of these processes such as incremental encoding processes and rule induction for each matrix were used by all participants, while other processes such as the induction of abstract relations of the dynamic management of different goals in memory were specific to participants with above-average performance. Although this model provides a strong theoretical explanation for individual differences in Raven performance, its application remains limited.

Cognitive measurement models may instead provide person- and condition-specific parameters for distinct cognitive processes. These person-specific parameters can be easily used as measures of individual differences in specific aspects of cognitive processes, which can then be related to performance in intelligence tests. For instance, parameters of the drift-diffusion model, that will be introduced later, have been associated with performance in intelligence test or memory tasks [26,50,51,52]. In this, parameters from cognitive measurement models may thus provide insights on which cognitive processes are actually linked to intelligence.

While all cognitive models are deliberate simplifications of the cognitive processes within a task and rely on often critically discussed assumptions, there is actually no alternative to the use of a measurement model, may it be statistical or cognitive. While most research does not explicitly decide for a specific measurement model, by calculating the mean performance for a person in a task (as often done) they implicitly adopt a statistical measurement model that makes no explicit statements about the underlying cognitive processes of the measurement. It may even be argued that not explicitly deciding for a specific measurement model is practically similar to implicitly using the most simple cognitive model at hand: A model assuming that the observed variable directly represents the cognitive processes of interest. As already mentioned earlier, this assumption is almost always false. Therefore, we would argue that using explicit measurement models is always superior to equating observed variables with the cognitive process of interest.

To convey an idea of the benefits of the application of cognitive modeling in intelligence research, we will discuss three examples of cognitive models in the following sections. We selected different models describing cognitive processes of particular interest to intelligence research, such as decision making, working memory, and cognitive control, and demonstrate how they may be used to quantify individual differences in the respective cognitive processes. Please note that the three models described below differ in their breadth of application and in their former use as explanatory or measurement model. Following these examples, we then provide guidelines for choosing the appropriate model for a particular research question.

### 3.1. Different Cognitive Models of Interest for Intelligence Research

#### 3.1.1. The Drift Diffusion Model of Binary Decision Making

**Figure 1:**
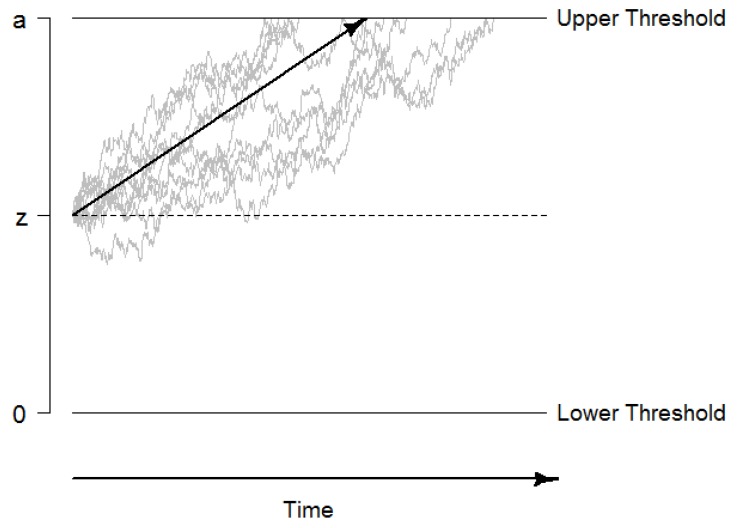
Graphical illustration of the drift-diffusion model. The decision process starts at the starting point *z*, and information is accumulated until the boundary *a* is reached. The systematic part of the accumulation process, the drift rate *v*, is illustrated with the black arrow. The non-decision time $t_{0}$ is not included in this figure.

The drift diffusion model (DDM) describes performance in two-alternative forced choice decisions tasks. The model assumes that evidence is accumulated in a random walk process until one of two decision thresholds is reached, the decision process is terminated, and a motor response (usually a key press) is initiated (see Figure 1 for an illustration; [47]). This evidence accumulation process can be described by a Wiener diffusion process that consists of a systematic component, the drift rate *v*, and normally distributed random noise with a mean of 0 and a variance of $s^{2}$ (this so-called diffusion constant *s* is usually fixed to a standardized value such as 0.1 or 1 for reasons of identifiability). The drift rate can be considered as a performance measure that directly quantifies the velocity of information uptake. In addition, the DDM quantifies the distance between decision thresholds as a measure of speed-accuracy trade-offs and decision cautiousness in the boundary separation as the parameter *a*, the starting point of evidence accumulation as the parameter *z*, and the time of non-decisional processes such as encoding and response preparation and execution as the parameter $t_{er}$ or $t_{0}$. Beyond these basic parameters, intra-individual variability parameters have been added to the DDM (i.e., $st_{0}$, sv, and sz) to account for inter-trial variability within a person [47,53].

The validity of DDM parameters has been demonstrated both by parameter recovery studies [54] and by experimental validation studies [55,56,57]. Moreover, model parameters have been showing satisfying reliabilities estimated with test-retest correlations given sufficiently large trial numbers [58] and at least drift rates have been shown to exhibit trait-like properties [59]. Specifically, Schubert et al. [59] used latent-state trait models with additional method factors [60] to separate different variance sources across three different tasks and two measurement occasions. The results showed that the variance consistent across tasks and measurement occasions was largest for drift rates (on average 44%), while this variance was considerably lower for boundary separations and non-decision times (between 32 to 36%). Although drift rates captured this amount of variance that was consistent across tasks and measurement occasions best, single task estimates of drift rates were only moderately reliable (Rel=0.38−0.69) and still contained considerable method specific variance (9 to 17%). Therefore, individual differences in drift rates should always be measured across different tasks if one is interested in individual differences in the underlying latent trait.

Altogether, it is not surprising that the DDM is the most frequently used cognitive model in intelligence research. By mathematically identifying parameters quantifying the speed of information uptake (*v*), the decision cautiousness (*a*), and encoding and movement times ($t_{er}$), it renders complicated experimental setups that have been used to dissociate these elements of the decision process with little success unnecessary [61]. Several studies have reported positive associations between cognitive abilities and drift rates e.g., [26,50,52,62,63,64], whereas the other model parameters have been shown to be largely unrelated to fluid intelligence [26,52,64]. The application of the DDM to data sets is made fairly easy by user-friendly software such as *EZ* [65,66] and *fast-dm* [67].

The DDM is part of a larger family of evidence accumulation models that provide a general description of decision processes. Another member of this model family is the linear ballistic accumulator model (LBA; [68]), which presumes that a number of independent accumulators race towards a common response threshold. Hence, where the DDM can only be applied to data from two-choice reaction times tasks, the LBA can be applied to data from both two- and multiple-choice reaction time tasks. Another member of this model family is the leaky, competing accumulator model (LCA; [69]), which entails a number of stochastic accumulators that compete against each other via mutual inhibition to reach a decision threshold. Both models have not been applied in intelligence research yet, probably because they do not provide a single performance measure such as the drift rate of the DDM, as one drift parameter for each of the accumulators is estimated in LBA and LCA models, resulting in several drift rates.

#### 3.1.2. The Time-Based Resource-Sharing Model of Working Memory

**Figure 2:**
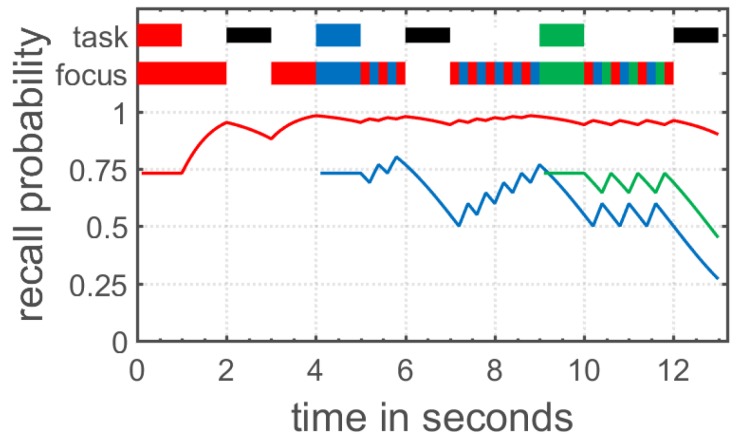
Visualization of the time-based resource sharing (TBRS) theory as implemented in the TBRS2 model by Gauvrit and Mathy [75]. At the top, the current task is displayed. A colored box represents a to- be-encoded memory item, a black box represents a distractor task, and a white box represents free time. Below, the focus of attention is shown. During free time, participants engage in refreshing of the already encoded memory item; during distractor tasks or encoding of other items, the already encoded memory items decay over time.

The time-based resource sharing (TBRS) model of working memory started out as a verbal theory explaining the performance in complex span tasks measuring working memory capacity [70,71], but has been extended to verbal and visual WM in general [72,73,74]. The TBRS model claims that processing and the maintenance of stored information rely on the same attentional resource in working memory. Because of this attentional bottleneck, only one of these two processes can be performed at a given time. In detail, the model assumes that information stored in working memory decays over time, unless this decay is counteracted by an attentional refreshing process or verbal rehearsal. Moreover, additional processing demands as imposed in complex span tasks shift attention towards these secondary processing tasks, resulting in the decay of items stored in working memory (see Figure 2 for an illustration). Altogether, working memory as conceptualized in the TBRS model continuously shares attentional resources between maintenance and processing in order to counteract decay of memory items and efficiently process information.

In recent years there have been formalizations of the TBRS model as an explanatory model [48] and as a simplified measurement model [75]. Such models may be of great interest for the field of intelligence research, not only because intelligence is strongly related to working memory [14,76,77], but because the field is still in debate about which specific cognitive processes within working memory, storage or executive processing, underlie its strong relationship with intelligence [11,12]. While the explanatory TBRS* model by Oberauer and Lewandowsky [48] is fairly complex and foremost an in-depth test for the experimental predictions of the TBRS theory, the TBRS2 implementation by Gauvrit and Mathy [75] provides a simplified version of the TBRS model and allows to estimate parameters that are directly linked to specific processes within the TBRS model. Such a model may provide person specific estimates of different processes in working memory, such as the encoding strength when an item is presented (i.e., the baseline β) or the speed of attentional refreshing (i.e., the refreshing rate *r*). These parameters may provide further information on which specific processes within working memory give rise to the strong relationship between working memory and intelligence.

As the mathematical implementations of the TBRS model have been developed only recently, there have not been any independent, systematic validation studies for the parameters of the model. Moreover, the psychometric properties of the model estimates (i.e., their reliability and validity) have not yet been assessed. Additionally, there is still a controversial debate in cognitive psychology whether decay actually is the core process limiting working memory capacity [78]. Although there are competing explanatory models of working memory questioning the role of decay as a limiting factor for working memory capacity [42,48], these models have not yet been translated into simple measurement models that allow estimating person-specific parameters of cognitive processes within working memory^1^. Until then, the TBRS2 model may provide a first step for including cognitive measurement models of working memory in intelligence research.

#### 3.1.3. The Shrinking Spotlight Model of Selective Attention

**Figure 3:**
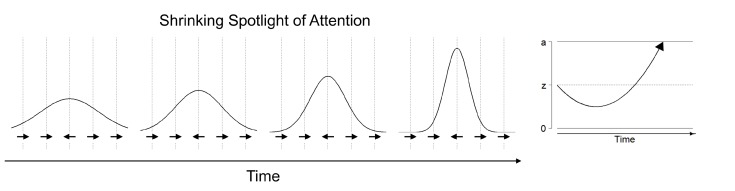
Illustration of the Shrinking Spotlight model for selective attention. The attentional focus narrows to the central arrow over time (**left part**). This results in a stronger weight of the critical information (i.e., the central stimulus) in the drift-rate of an associated diffusion process (**right part**).

The shrinking spotlight model of selective attention describes processing in the Eriksen flanker task, in which participants have to respond according to the orientation of a centrally presented target arrow while ignoring irrelevant arrows flanking the target stimulus [20,80]. The shrinking spotlight model is an extension of the drift diffusion model of sequential processing: It assumes that both target and flanker arrows provide perceptual evidence *p* for a particular response weighted by the amount of attention *a* allocated to each of these stimuli. The drift rate consists of the sum of weighted perceptual evidence across all stimuli at a given time. Over time, attention is assumed to zoom in on the central arrow, reflecting a narrowing of the focus of selective attention on the target stimulus. Thus, the target stimulus is weighted more strongly in comparison to the flanker stimuli and therefore affects the drift rate more strongly over time (see Figure 3). The initial width of attentional distribution is estimated in the attentional spotlight parameter $sd_{a}$, which reflects the standard deviation of a Gaussian distribution centered on the target stimulus, whereas the rate of attentional distribution reduction is estimated in the parameter $r_{d}$. In addition, the model also allows estimating the encoding and movement times in the $t_{er}$ parameter, and the distance of symmetrical response thresholds from the starting point of evidence accumulation in the parameters *A* and B=−A.

The model has been shown to be able to account for data from a standard flanker task and experimental manipulations of task properties have been shown to specifically affect single model parameters [80]. Moreover, parameter recovery studies have shown that model parameters can be accurately recovered with as few as only 50 experimental trials [81]. However, simulation results have also shown that the model is not able to recover the attentional spotlight and the shrinking rate parameter accurately, because a wide initial spotlight with a high shrinking rate makes the same predictions as a narrow initial spotlight with a low shrinking rate [81]. Therefore, it has been recommended to calculate a composite measure of the duration of interference as the ratio of the two parameters, $sd_{a}/d_{r}$, to account for the trade-off during model estimation. Although there have not yet been any systematic analyses on the psychometric properties of parameter estimates, correlations of r=0.42−0.80 between model parameters across different cue conditions of the Attention Network Test suggest at least moderate to good reliabilities, especially for the interference ratio with correlations of about r≈0.80 [82]. So far, the shrinking spotlight model has not yet been applied in intelligence research, but it would be promising to relate individual differences in the susceptibility to interference (as reflected in the interference parameter) to individual differences in intelligence test performance and working memory capacity to further explore the role of selective attention in cognitive abilities.

An alternative account of performance in the Eriksen flanker task is given by the dual-stage two phase model [83]. This model proposes two distinct processing stages: In the first processing stage, evidence accumulation is affected both by evidence accumulation towards the response associated with the target stimulus and by evidence accumulation towards the response associated with the flanker stimuli. At the same time, an attention-driven parallel evidence accumulation process selects a single stimulus for further processing. If this stimulus selection process terminates before response selection is finished, response selection enters a second stage with the drift rate being solely determined by the selected stimulus. As of yet, model comparison studies have not yet decided which of the two models provides the best account of selective attention phenomena [80,81,83,84]. Both models can be fit to data and subsequently be compared using the R package *flankr* [85].

### 3.2. Guidelines for Model Selection

**Figure 4:**
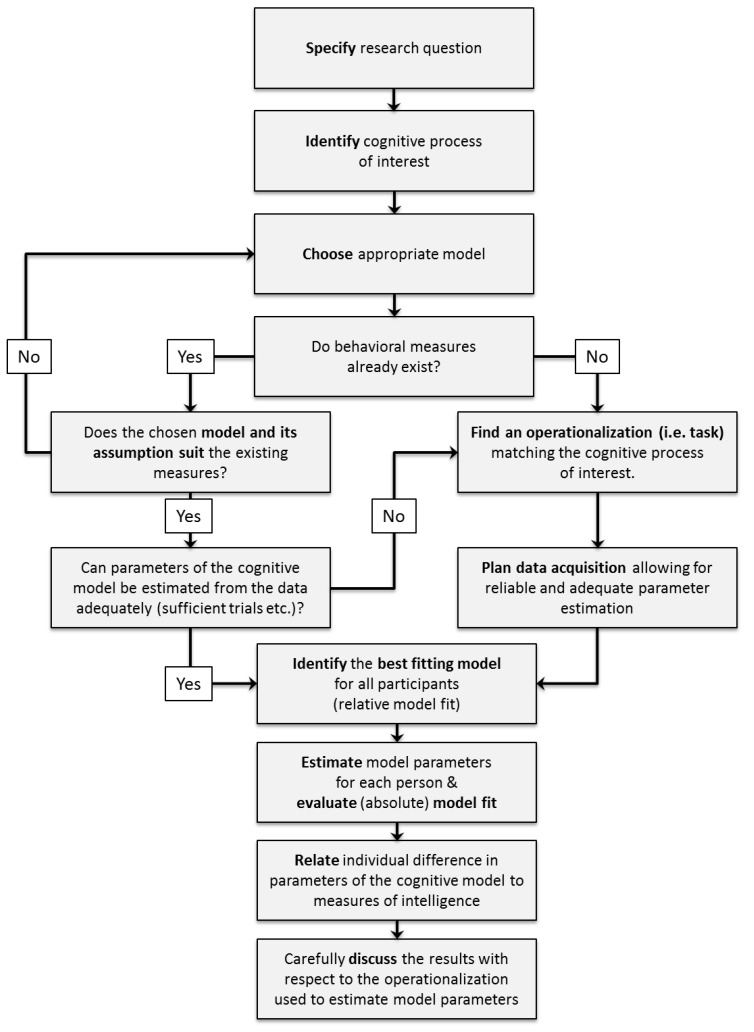
Flowchart illustrating the different planning and decision steps when using cognitive models in intelligence research.

When deciding which cognitive model to use for a specific research question, there are some conceptual and practical issues to be considered in order to select the appropriate model: First of all, the research question has to be specified. Second, the cognitive processes of interest that are to be related to general intelligence for this research question have to be identified. Third, an appropriate model providing a description of these cognitive processes has to be chosen. During this step, theoretical reasons for choosing one model over its alternatives should be considered. Fourth, experimental tasks congruent with the assumptions of the selected model should be selected to allow the valid estimation of model parameters. For an illustration of these decision steps see the upper part of Figure 4 (p. 10).

In general, discussing these issues during project planning aims to strengthen two important points for the conclusions from the modeling results. On the one hand, researchers should clarify which specific cognitive processes they are interested in and select a cognitive model accordingly. On the other hand, researchers should maximize the fit between the measurement or operationalization of a specific cognitive process (i.e., the task used) and the selected cognitive model.

For example, a group of researchers might be interested in which cognitive processes in simple decision tasks are related to intelligence. Such tasks may require participants to decide whether a number is odd or even, or whether a letter is a vowel or consonant. They decide to use the drift diffusion model to quantify the different cognitive processes associated with binary decision making. However, one of these tasks has an additional switching demand, requiring participants to switch between the number and the letter decision (for an example, see [86]). Because this task is a binary decision task, the drift-diffusion model may still provide suitable estimates for the cognitive processes in such a task [87,88]. However, this task arguably requires more than one decision: On the one hand the decision which task is to be carried out, and on the other hand the decision corresponding to the task. Thus, this task does not fully fit the conceptualization of the drift-diffusion model as there may not be a single decision process but two. Therefore, researchers should either think about using a different task that has a better fit to the basic assumptions of the drift-diffusion model or search for an alternative model that better fits the task they want to use.

This example reiterates the importance of an explicit and critical decision for a specific cognitive measurement model with respect to the measurement and operationalization that has already been pointed out before. As the developers of cognitive models often suggest a specific task suitable for parameter estimation e.g., [75], the initial model publication is usually a good starting point for finding prototypical tasks that match the model assumptions. For popular cognitive models such as the diffusion model there are review articles summarizing studies in which the diffusion model was successfully applied to data from several different tasks [55]. Although some of these prototypical tasks may not provide the most suitable measures for a specific research question, they nevertheless constitute a meaningful starting point.

## 4. Guidelines for Model Application

After identifying an appropriate model based on theoretical considerations as outlined in the previous section, we strongly recommend to further plan the application of mathematical models ahead of data collection to ensure the interpretability and trustworthiness of the estimated model parameters. Specifically, three basic steps should be pursued when applying a cognitive model to a specific research question (see lower part of Figure 4, p. 10):

1. Researchers should plan their data collection to meet requirements for reliable and stable parameter estimates.
2. Model fit should be carefully evaluated after fitting the model to the empirical data.
3. Model parameters should be adequately related to other individual differences variables of interest such as intelligence test performances.

In the following section, we will provide step-by-step instructions using examples from the application of diffusion models in intelligence research, which may serve as guidelines when using any kind of cognitive model in individual differences research.

### 4.1. Design and Data Collection

#### 4.1.1. Reliability and Stability of Estimated Model Parameters

The reliable estimation of model parameters from empirical data usually requires more data points than would be needed if only applying a statistical model to the data. For illustration, compare the description of reaction time distributions in decision tasks by a Gaussian distribution to the description by a diffusion model. When describing performance in a binary choice task by a Gaussian distribution, 20–30 trials are usually sufficient to provide reliable estimates of means and standard errors of the distribution [89]. When describing performance by a diffusion model, however, many more trials are needed because model parameters are not calculated analytically, but are found by fitting them to empirical response time distributions in an iterative process. Hence, a small number of trials will result in an inadequate representation of the full response time distribution and will therefore impair the estimation of model parameters describing distributional elements beyond measures of central tendency [90].

For the basic DDM (with the four parameters drift rate, boundary separation, starting point, and non-decision time), simulation studies have shown that 100 trials are sufficient to produce relatively reliable estimates of drift rates and that no further increases in parameter reliabilities are gained by increasing trial numbers beyond 500 trials [90]. For other measurement models less prominently used in individual differences research, such systematic simulation studies have not yet been conducted. Therefore, we urge researchers interested in applying less frequently used models to run a simulation study before starting data collection to determine how many experimental trials are needed for a reliable parameter recovery. While a simulation does not guarantee reliable parameter estimates for an experiment in general, it rules out that low reliability is due to noisiness in the parameter estimation process.

#### 4.1.2. Trait, Situation, and Task Characteristics of Model Parameters

In addition, it is important to consider to what degree individual differences in model parameters reflect individuals’ personality traits or abilities, and to what degree they reflect task-specific characteristics, state-specific characteristics, and unsystematic measurement error. Imagine applying a model of verbal working memory to complex span data: Model parameters such as the individual rate of verbal refreshing or the ability to resist interference from distracting stimuli would reflect both individuals’ *general abilities* in verbal refreshing and inhibition of interference as well as their abilities to maintain memory stimuli *in this specific task*. Depending on the research question, researchers may be more interested in the general ability to maintain information in working memory as reflected in those parameters across different working memory tasks, or they may be interested in the specific ability to maintain information in working memory in precisely this task.

Usually, intelligence research questions are more likely to concern abilities generalized across specific operationalizations and situations than abilities in specific operationalizations or situations. However, model parameters estimated in a specific task are always going to contain both trait-, state- and task-specific amounts of variance [60,91]. For example, a latent state-trait analysis of DDM parameters in elementary cognitive tasks revealed that only about 45 percent of the variance in task-specific drift rates was accounted for by the common trait, and that only about 30 to 35 percent of the variance in task-specific boundary separation and non-decision time parameters were accounted for by their respective common traits [59]. Therefore, if a research question using cognitive models in intelligence research concerns performance in certain cognitive processes that is generalizable across specific operations, it may be worthwhile to design a test battery consisting of three or more tasks to which the cognitive model can be applied. Averaged or latent performance in process parameters across tasks will then allow a more precise estimate of individuals’ performance in model parameters that is independent of specific task or situation characteristics.

### 4.2. Evaluation of Model Fit

#### 4.2.1. Relative Model Fit: Which Model Provides the Best Account for the Data?

After finishing data collection, but before relating model parameters to intelligence tests or other covariates, it is necessary to evaluate how well a chosen model describes the empirical data and to possibly adjust model specifications to increase model fit. For most cognitive models, these empirical data consist of single-trial accuracies and/or response times, but aggregate measures such as proportion correct for different conditions might also be entered into the analysis. Before the raw data are entered into any kind of model, they should be carefully inspected for extreme values or other distributional properties that violate model assumptions and that may impair or even systematically bias parameter estimation. Once fidelity in these raw values has been established, cognitive models can be fitted to these empirical data. For this purpose, it has to be decided how many and which model parameters will be estimated and which model parameters will be fixed, because they are not expected to be affected by task characteristics or are not of interest for the current research question. Moreover, if experimental tasks contain several conditions, it may be necessary to decide which (if any) parameters are allowed to vary between conditions. It may even be desirable to split data from different conditions into separate data sets for separate model estimations to be able to subsequently model these separately estimated model parameters as latent variables. For this purpose, it may be helpful to reflect on the relationship between model complexity and the stability of parameter estimates: The more parameters of a model are estimated, the more likely it is to provide an accurate account of the data. However, if too many model parameters are estimated relative to the number of experimental trials, the stability of parameter estimates will be impaired [90,92].

Therefore, we suggest fitting several models to the empirical data containing different combinations of estimated or fixed parameters that are consistent with the current research question, unless there are strong theoretical reasons to decide on a specific model instantiation a priori. These models can then be compared based on parsimonious fit indices such as the Akaike Information Criterion (AIC; [93]) or the Bayesian Information Criterion (BIC; [94]), which take into account both model fit and model parsimony, to identify the model making the best trade-off between model fit and model complexity. As mentioned before, this model comparison step may not be necessary when a priori deciding for a specific instantiation of the model.

However, this model comparison approach only addresses one element of model fit evaluation, *relative model fit*. By identifying the best-fitting specification of the model out of a number of alternative specifications, it is possible to identify the model providing the best description of the empirical data. However, this does not guarantee that the best-fitting model provides a *good* description of the data.

#### 4.2.2. Absolute Model Fit: How Well Does the Selected Model Describe the Data?

Therefore, in the next step the *absolute model fit* has to be evaluated to decide if the model can be accepted for all data sets. Absolute model fit is typically ascertained by either (a) statistical tests of model fit, (b) goodness-of-fit (GOF) indices, or (c) graphical inspections of model fit.

Statistical tests of model fit quantify the discrepancy between the empirical data and model predictions by means of a test statistic that is then tested for significance. However, this null hypothesis-testing of model fit contains several problems, as the power of statistical tests is closely tied to the amount of data available. When only a few trials are available, statistical tests may not be capable of rejecting the null hypothesis due to a lack of power, whereas when the trial number is large, statistical tests tend to become overly sensitive and detect even irrelevant deviations between the empirical data and model predictions [95]. To overcome some of the problems associated with null hypothesis testing, it has been suggested to simulate a large number of data sets based on the estimated model parameters, fit the model to each of the simulated data sets, and derive the 95 percent or 90 percent quantile of the resulting distribution of *p*-values as a critical value for the statistical tests of the originally estimated models [96,97]. However, models will still be accepted with an unknown error probability.

Goodness-of-fit indices are much more common in individual differences research, where they are used to evaluate the model fit of structural equation models [98], than in cognitive modeling. GOF indices standardize test statistics and take into account both model complexity and the number of data points. Typically, GOF indices have a fixed value range from 0 to 1 with certain cut-off values that indicate acceptable or good model fit. GOF indices are less frequently used in cognitive modeling, probably because several GOF indices used in structural equation modeling require the comparison of the actual model to a minimally plausible baseline model, which cannot be easily specified for most cognitive models. However, it has been recently suggested to adapt the root mean square error of approximation for the evaluation of cognitive models that can be fitted with a $\chi^{2}$-distribution, such as the diffusion model [95]. Note that simulation studies have shown that this approach is only advisable when trial numbers are sufficiently large.

Finally, a third and widespread approach to the evaluation of absolute model fit is to graphically compare the empirical data to model predictions. To graphically inspect model fit, empirical data can be plotted against or overlaid by model predictions separately for each participant or aggregated over participants. This process can be rather time-consuming in larger samples if each participant is inspected individually. Moreover, it is important to be aware of the fact that graphical evaluations of model fit are inherently subjective and may therefore lead to spurious conclusions [99]. Having two independent raters evaluate model fit and discuss their conclusions may therefore increase the objectivity of the evaluation process.

If individual data sets can be identified that do not provide a satisfying model fit, raw data should be inspected for coding errors or outliers that may need to be removed (e.g., extremely fast reaction times with decision behavior close to guessing in a decision task). If model fit remains unacceptable, individual data sets may then need to be removed from further analyses, as it cannot be ascertained that the model parameters characterize the cognitive processes in the task accurately.

### 4.3. Relating Model Parameters to Intelligence Test Performance

Finally, after reliable estimates for the best fitting model have been obtained, the model parameters should be related to measures of intelligence. While this seems straightforward, there are actually two major methodological concerns.

First, extreme values in either parameter estimates or cognitive abilities measures need to be addressed. If extreme values (univariate outliers) are detected in parameter estimates, it is imperative to inspect if any outliers, coding errors, or abnormal distributional properties of the participant’s raw data may have contaminated parameter estimation. If this is the case and if these outliers only constitute only a small amount of the data, they should be removed or winsorized and the model fitting procedure should be repeated to see if this treatment has led to more reasonable parameter estimates. If parameter estimates are still extreme or if outliers in raw data cannot be dealt with (e.g., because this participant’s distribution of raw data deviates from model assumptions), this participant should be removed from further analyses as model parameters most likely reflect other properties of cognitive processes for this participant than for the rest of the sample. A similar problem is raised by multivariate outliers that may need to be removed based on an inspection of scatterplots or the calculation of the Mahalanobis distance. It goes without saying that information about the number of data points and/or participants removed and a rationale of their removal needs to be included in any description of the modeling results.

Second, researchers usually obtain one or more person-specific estimates for each model parameter of interest across different tasks or experimental conditions, just like they do when using aggregated performance measures such as accuracies or mean reaction times. Then the relationship of these model parameters with intelligence test scores is estimated by means of correlations or structural equation modeling. However, this approach represents a sequential analysis plan that treats the estimated parameters as manifest variables when quantifying the relationship between parameters of the cognitive model and the intelligence measures.

Treating estimated model parameters as manifest variables ignores the uncertainty that these parameters inherit from estimation and leads to an underestimation of standard errors in the second analysis step [100]. In fact, this is the case both for behavioral aggregates, such as mean reaction times or proportion correct, and for model parameters that are estimated from behavioral data or calculated from aggregate performance measures. Although this does not necessarily affect the estimated size of the relation between parameters obtained from cognitive models and intelligence measures, a sequential analysis plan always leads to an overestimation of the statistical significance of the estimated relationships [101].

A solution to this problem is hierarchical modeling [102,103]^2^. In hierarchical modeling approaches, parameters of a cognitive model can be estimated simultaneously not only for all participants but across various tasks. Additionally, relationships with third variables, such as intelligence, can be estimated in the same step. On the one hand, such models avoid underestimating the standard errors of the relationship between model parameters and third variables such as intelligence measures by simultaneously estimating the model parameters and their relationship to intelligence (for an example of hierarchical models of the worst performance rule, see [101]). On the other hand, by assuming that the distribution of model parameters across individuals follows a higher order distribution^3^ (so called hyper-priors), hierarchical models do not estimate parameters for each individual independently, but instead estimate model parameter for each individual informed by the parameter estimates from all other individuals. Not only does this render the parameter estimation more robust, but it also allows obtaining reliable estimates for the parameters of a cognitive model for each individual with fewer trials (for an example, see the hierarchical diffusion model: [105]).

Although this modeling approach is structurally similar to hierarchical modeling in latent variable models (i.e., SEM), there are some important differences. While hierarchical latent variable models separate general from specific factors in between person variances e.g., [8], hierarchical models in the field of cognitive models distinguish between parameters estimated within a person and the distribution of parameters between persons. In this, hierarchical modeling of cognitive processes is closely related to multi-level modeling separating the within and between person level [106].

For instance, when applying the diffusion model, parameters of each individual can be estimated independently without assuming a specific distribution of estimated model parameters across participants. While this achieves the highest flexibility in parameter estimation, this approach ignores possible information from the between person level. In contrast, hierarchical modeling assumes that the parameters from each individual stem from a distribution of parameters on the between person level, and thus parameters for each individual are estimated taking information from all other subjects into account. As stated before, this account has two important benefits: (1) hierarchal modeling renders the parameter estimation for each individual more efficient [105]; and (2) parameters and their relationship to third variables like intelligence can be estimated simultaneously, accounting for the uncertainty of parameter estimates and thus adequately reporting the significance of the relationship between parameter estimates and third variables [101].

A serious complication of hierarchical modeling is that these models typically have to be explicitly specified and translated into code for each application, and that software solutions for parameter estimation are still rare. Nevertheless, hierarchical models do provide the mathematically accurate and sound solution for estimating the relationship between estimated model parameters and intelligence measures. Still, the sequential estimation of model parameters and their relationship to intelligence test scores seems to yield results comparable to hierarchical approaches [101]. In conclusion, while sequential approaches may overestimate the statistical significance of the relationship between model parameters and covariates (biasing inference), they nevertheless provide reasonable and unbiased estimates of the effect size of this relationship. For the future, it would be desirable that the application of cognitive modeling in the field of intelligence research or individual differences in general leads to the development of further simple software solutions or R packages [107,108] that simplify the use of hierarchical models.

## 5. Interpretation of the Results

Regardless of how the relationship between parameters from a cognitive model and intelligence measures is estimated, ultimately this relationship has to be interpreted on a conceptual level. Although parameters of a cognitive model provide more specific information about the cognitive process underlying the behavioral responses, these parameters still have to be interpreted with respect to the operationalization of the cognitive process. For instance, the diffusion model can be estimated in a broad set of tasks, ranging from perceptual judgment tasks (e.g., a random-dot motion task), over elementary cognitive tasks (e.g., Posner or Sternberg task), to even more complex memory tasks. In all of these different tasks, the diffusion model estimates the same set of parameters (i.e., drift rates, boundary separations, and non-decision times). However, this alone does not imply that model parameters estimated in the different tasks can be interpreted the same way. Specifically, the drift rate estimated in a random-dot motion task may represent the speed of perceptual information accumulation towards one response alternative. In a memory recognition task, however, the drift rate would rather be interpreted as the signal-to-noise ratio of the representation in memory. Beyond a theoretical discussion of the similarity of different tasks, statistical methods such as factor analysis or structural equation models can be used to get further information on variance that is shared across different tasks, or that is specific to a task or a situation (see: [59]). However, all in all, the interpretation of parameters of a cognitive model always relies on the specific experimental tasks.

In general, a cognitive model always represents a structural description of the behavioral measures from a specific task. The semantic meaning of the parameters of a model, however, can only be obtained with respect to the context (i.e., the task or materials) they are estimated in. Consider the following equation: v=x/t. On its own, this equation is merely a structural description how *v* can be obtained from *x* and *t*. In contrast, if the context of the observations of *x* as a distance between two points, and *t* as the time taken to get from one point to the other is known, then *v* can reasonably interpreted as the average speed of travel. It is just the same with parameters from any cognitive model: Without the context of their estimation they are merely transformations or estimated simplifications of the observed variables. Adding the semantical context of the observations however allows to interpret the parameters in a meaningful way.

All in all, the matter of adequately interpreting parameters of a cognitive model relates to a broader issue, namely validity. On the one hand, there is the question of how far a cognitive model provides a valid description of the cognitive process underlying the behavioral responses in a task. On the other hand, there is the question of how far individual differences in these parameters can be generalized across different tasks and assumed to represent between person variation in a more general and task-unspecific cognitive process. These are hardly problems that can be solved within a single study, but there is a combined effort needed to establish which parameters of cognitive models provide meaningful representations of individual differences in specific aspects of cognitive processing.

For example, attempts to unite psychophysiological and neuroimaging research with cognitive modeling may be particularly informative about issues of validity, as they allow a direct test of the idea that process parameters reflect certain neural correlates. Several studies have already suggested a close link between diffusion model parameter and neural processing correlates in the EEG. In particular, the latency of the N2, which is a neural correlate of visual encoding time, has been shown to be associated with the non-decision time parameter of the diffusion model [109], and the buildup rate of a positive centroparietral positive potential has been suggested to directly reflect the rate of evidence accumulation captured in the drift rate parameter on a neural level [110,111].

Altogether, following certain guidelines and carefully discussing the underlying assumptions and the operationalization when using a cognitive model provides a more explicit approach to measuring individual differences in cognitive processes, and thus represents a decisive improvement compared to the prevailing methods. At the very least, such careful reflection might immunize against the category error that cognitive models are accurate reflections of a latent, unobservable cognitive process. Any (cognitive) model cannot be anything but a simplification of reality that, if successful, captures the most important aspects, but never the entirety of an ontological (cognitive) process. In the same way that a map provides a simplification of a city’s layout that is useful for navigation without ever providing a detailed account of the whole city system, cognitive models may refine our understanding of how those unobservable cognitive processes operate and thereby facilitate the measurement of certain process properties. Or, as Box [112] put it: “All models are wrong, but some models are useful”.

## 6. Conclusions

Altogether, incorporating cognitive models in intelligence research provides numerous advantages. On the one hand, cognitive models provide explicit theoretical descriptions of cognitive processes that may underlie individual differences in general intelligence. On the other hand, they allow to estimate person specific parameters for each individual that can be related to measures of intelligence. Therefore, cognitive models allow to relate theoretically founded measures of individual differences in parameters of cognitive processes to individual differences in general intelligence and to overcome the fuzzy theoretical interpretation of behavioral indicators such as reaction times or accuracies.

Beyond that, cognitive models may allow identifying the effects of experimental or pharmacological interventions and training interventions on specific cognitive processes. For example, the shrinking spotlight model of selective attention might be used to test if a training intervention aimed at improving selective attention actually affects interference parameters of the model or if the intervention only reduces non-decision times or response thresholds. In a similar vein, the drift diffusion model might be used to characterize experimental effects of a pharmacological intervention on mental speed by distinguishing an increase in the velocity of evidence accumulation from an increase in motor response times. Last but not least, cognitive process parameters could not only be related to general intelligence differences, but also to individual differences in neural measures related to cognitive abilities, and may thus provide a different and possibly more complete perspective on the neuro-cognitive processes giving rise to individual differences in general intelligence. Taken together, the application of cognitive models as elaborate measurement tools provides an exciting new avenue for research on the neuro-cognitive processes underlying intelligence.

This approach focuses on insights into the cognitive correlates of general intelligence and does not represent an actual theory of general intelligence. On the one hand, it shows that developing cognitive models for specific cognitive processes is possible. On the other hand, proper theories of general intelligence that provide a comprehensive and mechanistic description of general intelligence as a cognitive process are scarce. As long as *theories* of general intelligence are mainly concerned with its factorial structure (i.e., psychometric theories, [2,5,6,7,8]), developing a cognitive model of general intelligence in the sense of a process theory remains difficult. One recently published positive counterexample is process-overlap theory, which suggests that the positive manifold may arise from a set of various domain-specific and domain-general cognitive processes which are linked multiplicatively [4]. In conjunction with mathematical models of the cognitive processes involved in process-overlap theory, this conceptual idea might be used to develop a formal model from different cognitive models that are linked in the multiplicative way suggested in process-overlap theory. In this sense, integrating mathematical models of cognitive processes that are correlated with measures of intelligence may provide a first step towards a comprehensive process theory of general intelligence—something for which the field has been searching for a long time.

## Footnotes

1. Oberauer and Lewandowsky [79] are working on an alternative measurement model that is more closely connected to interference models of working memory [42,45]. For a preprint, see: osf.io/vkhmu.

2. These two references focus on Bayesian hierarchical modeling. While Bayesian parameter estimation might have additional advantages over frequentist estimation approaches [104], the benefits of hierarchical modeling apply to both Bayesian and frequentist methods.

3. Typically a Gaussian distribution with a mean and standard deviation is assumed.

## Abbreviations

The following abbreviations are used in this manuscript:

SOB-CS	Serial-Order in Box Model for Complex Span Tasks
DDM	Drift-Diffusion Model
LBA	Linear Ballistic Accumulator Model
LCA	Leaky Competing Accumulator Model
TBRS	Time-base resource sharing theory/model
AIC	Akaike Information Criterion
BIC	Bayesian Information Criterion
GOF	Goodness-of-fit

## References

[1] De Boeck P. (2013). Intelligence, Where to Look, Where to Go?. J. Intell..

[2] Thurstone L. (1938). Primary Mental Abilities.

[3] Kievit R.A., Davis S.W., Griffiths J., Correia M.M., Henson R.N. (2016). A watershed model of individual differences in fluid intelligence. Neuropsychologia.

[4] Kovacs K., Conway A.R.A. (2016). Process Overlap Theory: A Unified Account of the General Factor of Intelligence. Psychol. Inquiry.

[5] Spearman C. (1904). ‘General intelligence’, objectively determined and measured. Am. J. Psychol..

[6] Horn J.L., Cattell R.B. (1966). Refinement and test of the theory of fluid and crystallized general intelligences. J. Educ. Psychol..

[7] Carroll J.B. (1993). Human Cognitive Abilities: A Survey of Factor-Analytic Studies.

[8] McGrew K. (2005). The Cattell-Horn-Carroll Theory of Cognitive Abilities: Past, Present, and Future. Contemporary Intellectual Assessment: Theories, Tests, and Issues.

[9] Jensen A.R. (2006). Clocking the Mind: Mental Chronometry and Individual Differences.

[10] Kyllonen P.C., Zu J. (2016). Use of Response Time for Measuring Cognitive Ability. J. Intell..

[11] Colom R., Abad F.J., Ángeles Quiroga M., Shih P.C., Flores-Mendoza C. (2008). Working memory and intelligence are highly related constructs, but why?. Intelligence.

[12] Conway A.R., Cowan N., Bunting M.F., Therriault D.J., Minkoff S.R. (2002). A latent variable analysis of working memory capacity, short-term memory capacity, processing speed, and general fluid intelligence. Intelligence.

[13] Engle R.W., Tuholski S.W., Laughlin J.E., Conway A.R. (1999). Working memory, short-term memory, and general fluid intelligence: A latent-variable approach. J. Exp. Psychol. Gen..

[14] Kyllonen P.C., Christal R.E. (1990). Reasoning ability is (little more than) working-memory capacity?. Intelligence.

[15] Unsworth N., Engle R.W. (2006). Simple and complex memory spans and their relation to fluid abilities: Evidence from list-length effects. J. Mem. Lang..

[16] Unsworth N., Engle R.W. (2007). The nature of individual differences in working memory capacity: Active maintenance in primary memory and controlled search from secondary memory. Psychol. Rev..

[17] Miyake A., Friedman N.P., Emerson M.J., Witzki A.H., Howerter A. (2000). The unity and diversity of executive functions and their contributions to complex ‘frontal lobe’ tasks: A latent variable analysis. Cognit. Psychol..

[18] Wongupparaj P., Kumari V., Morris R.G. (2015). The relation between a multicomponent working memory and intelligence: The roles of central executive and short-term storage functions. Intelligence.

[19] Thomson G.H. (1916). A hierarchy without a general factor. Br. J. Psychol. 1904–1920.

[20] Eriksen B.A., Eriksen C.W. (1974). Effects of noise letters upon the identification of a target letter in a nonsearch task. Percept. Psychophys..

[21] Stroop J.R. (1935). Studies of interference in serial verbal reactions. J. Exp. Psychol..

[22] Kane M.J., Meier M.E., Smeekens B.A., Gross G.M., Chun C.A., Silvia P.J., Kwapil T.R. (2016). Individual differences in the executive control of attention, memory, and thought, and their associations with schizotypy. J. Exp. Psychol. Gen..

[23] McVay J.C., Kane M.J. (2012). Why does working memory capacity predict variation in reading comprehension? On the influence of mind wandering and executive attention. J. Exp. Psychol. Gen..

[24] Donders F. (1969). On the speed of mental processes. Acta Psychol..

[25] Friston K.J., Price C.J., Fletcher P., Moore C., Frackowiak R.S., Dolan R.J. (1996). The trouble with cognitive subtraction. NeuroImage.

[26] Schubert A.L., Hagemann D., Voss A., Schankin A., Bergmann K. (2015). Decomposing the relationship between mental speed and mental abilities. Intelligence.

[27] Cronbach L.J., Furby L. (1970). How we should measure change: Or should we?. Psychol. Bull..

[28] Hedge C., Powell G., Sumner P. (2017). The reliability paradox: Why robust cognitive tasks do not produce reliable individual differences. Behav. Res. Methods.

[29] Borsboom D. (2005). Measuring the Mind: Conceptual Issues in Contemporary Psychometrics.

[30] Lord F., Novick M., Birnbaum A. (1968). Statistical Theories of Mental Test Scores.

[31] Schmidt F.L., Hunter J.E. (1999). Theory testing and measurement error. Intelligence.

[32] Borsboom D. (2008). Latent variable theory. Meas. Interdiscip. Res. Perspect..

[33] Borsboom D., Mellenbergh G.J. (2002). True scores, latent variables and constructs: A comment on Schmidt and Hunter. Intelligence.

[34] Schwarz W. (2001). The ex-Wald distribution as a descriptive model of response times. Behav. Res. Methods Instrum. Comput..

[35] Schwarz W. (2002). On the Convolution of inverse Gaussian and exponential Random Variables. Commun. Stat. Theory Methods.

[36] Miller R., Scherbaum S., Heck D.W., Goschke T., Enge S. (2018). On the Relation Between the (Censored) Shifted Wald and the Wiener Distribution as Measurement Models for Choice Response Times. Appl. Psychol. Meas..

[37] Keats J.A., Lord F.M. (1962). A theoretical distribution for mental test scores. Psychometrika.

[38] Wilcox R.R. (1978). Estimating true score in the compound binomial error model. Psychometrika.

[39] Matzke D., Wagenmakers E.J. (2009). Psychological interpretation of the ex-Gaussian and shifted Wald parameters: A diffusion model analysis. Psychon. Bull. Rev..

[40] Farrell S., Lewandowsky S. (2018). Computational Modeling of Cognition and Behavior.

[41] Heathcote A., Brown S., Wagenmakers E., Forstmann B., Wagenmakers E. (2015). An Introduction to Good Practices in Cognitive Modeling. An Introduction to Model-Based Cognitive Neuroscience.

[42] Oberauer K., Lin H.Y. (2017). An interference model of visual working memory. Psychol. Rev..

[43] Zhang W., Luck S.J. (2008). Discrete fixed-resolution representations in visual working memory. Nature.

[44] Banks W.P. (1970). Signal detection theory and human memory. Psychol. Bull..

[45] Oberauer K., Lewandowsky S., Farrell S., Jarrold C., Greaves M. (2012). Modeling working memory: An interference model of complex span. Psychon. Bull. Rev..

[46] Bröder A., Schütz J. (2009). Recognition ROCs are curvilinear—Or are they? On premature arguments against the two-high-threshold model of recognition. J. Exp. Psychol. Learn. Mem. Cognit..

[47] Ratcliff R. (1978). A theory of memory retrieval. Psychol. Rev..

[48] Oberauer K., Lewandowsky S. (2011). Modeling working memory: A computational implementation of the Time-Based Resource-Sharing theory. Psychon. Bull. Rev..

[49] Carpenter P.A., Just M.A., Shell P. (1990). What one intelligence test measures: A theoretical account of the processing in the Raven Progressive Matrices Test. Psychol. Rev..

[50] Schmiedek F., Oberauer K., Wilhelm O., Süß H.M., Wittmann W.W. (2007). Individual differences in components of reaction time distributions and their relations to working memory and intelligence. J. Exp. Psychol. Gen..

[51] Ratcliff R., Schmiedek F., McKoon G. (2008). A diffusion model explanation of the worst performance rule for reaction time and IQ. Intelligence.

[52] Schmitz F., Wilhelm O. (2016). Modeling Mental Speed: Decomposing Response Time Distributions in Elementary Cognitive Tasks and Correlations with Working Memory Capacity and Fluid Intelligence. J. Intell..

[53] Ratcliff R., Tuerlinckx F. (2002). Estimating parameters of the diffusion model: Approaches to dealing with contaminant reaction times and parameter variability. Psychon. Bull. Rev..

[54] Van Ravenzwaaij D., Oberauer K. (2009). How to use the diffusion model: Parameter recovery of three methods: EZ, fast-dm, and DMAT. J. Math. Psychol..

[55] Ratcliff R., McKoon G. (2008). The diffusion decision model: Theory and data for two-choice decision tasks. Neural Comput..

[56] Voss A., Rothermund K., Voss J. (2004). Interpreting the parameters of the diffusion model: An empirical validation. Mem. Cognit..

[57] Lerche V., Voss A. (2017). Experimental validation of the diffusion model based on a slow response time paradigm. Psychol. Res..

[58] Lerche V., Voss A. (2017). Retest reliability of the parameters of the Ratcliff diffusion model. Psychol. Res..

[59] Schubert A.L., Frischkorn G.T., Hagemann D., Voss A. (2016). Trait Characteristics of Diffusion Model Parameters. J. Intell..

[60] Steyer R., Schmitt M., Eid M. (1999). Latent state–trait theory and research in personality and individual differences. Eur. J. Personal..

[61] Longstreth L.E. (1984). Jensen’s reaction-time investigations of intelligence: A critique. Intelligence.

[62] Ratcliff R., Thapar A., McKoon G. (2010). Individual differences, aging, and IQ in two-choice tasks. Cognit. Psychol..

[63] Ratcliff R., Thapar A., McKoon G. (2011). Effects of aging and IQ on item and associative memory. J. Exp. Psychol..

[64] Schulz-Zhecheva Y., Voelkle M.C., Beauducel A., Biscaldi M., Klein C. (2016). Predicting Fluid Intelligence by Components of Reaction Time Distributions from Simple Choice Reaction Time Tasks. J. Intell..

[65] Wagenmakers E.J., Van Der Maas H.L.J., Grasman R.P.P.P. (2007). An EZ-diffusion model for response time and accuracy. Psychon. Bull. Rev..

[66] Wagenmakers E.J., van der Maas H.L.J., Dolan C.V., Grasman R.P.P.P. (2008). EZ does it! Extensions of the EZ-diffusion model. Psychon. Bull. Rev..

[67] Voss A., Voss J. (2007). Fast-dm: A free program for efficient diffusion model analysis. Behav. Res. Methods.

[68] Brown S.D., Heathcote A. (2008). The simplest complete model of choice response time: Linear ballistic accumulation. Cognit. Psychol..

[69] Usher M., McClelland J.L. (2001). The time course of perceptual choice: The leaky, competing accumulator model. Psychol. Rev..

[70] Barrouillet P., Bernardin S., Camos V. (2004). Time Constraints and Resource Sharing in Adults’ Working Memory Spans. J. Exp. Psychol. Gen..

[71] Barrouillet P., Bernardin S., Portrat S., Vergauwe E., Camos V. (2007). Time and cognitive load in working memory. J. Exp. Psychol. Learn. Mem. Cognit..

[72] Vergauwe E., Barrouillet P., Camos V. (2009). Visual and spatial working memory are not that dissociated after all: A time-based resource-sharing account. J. Exp. Psychol. Learn. Mem. Cognit..

[73] Vergauwe E., Camos V., Barrouillet P. (2014). The impact of storage on processing: How is information maintained in working memory?. J. Exp. Psychol. Learn. Mem. Cognit..

[74] Barrouillet P., Portrat S., Camos V. (2011). On the law relating processing to storage in working memory. Psychol. Rev..

[75] Gauvrit N., Mathy F. (2018). Mathematical transcription of the ‘time–based resource sharing’ theory of working memory. Br. J. Math. Stat. Psychol..

[76] Colom R., Rebollo I., Palacios A., Juan-Espinosa M., Kyllonen P.C. (2004). Working memory is (almost) perfectly predicted by g. Intelligence.

[77] Gignac G.E. (2007). Working memory and fluid intelligence are both identical to g? Reanalyses and critical evaluation. Psychol. Sci..

[78] Oberauer K., Farrell S., Jarrold C., Lewandowsky S. (2016). What limits working memory capacity?. Psychol. Bull..

[79] Oberauer K., Lewandowsky S. Simple Measurement Models for Complex Working-Memory Tasks. https://osf.io/vkhmu/.

[80] White C.N., Ratcliff R., Starns J.J. (2011). Diffusion models of the flanker task: Discrete versus gradual attentional selection. Cognit. Psychol..

[81] White C.N., Servant M., Logan G.D. (2018). Testing the validity of conflict drift-diffusion models for use in estimating cognitive processes: A parameter-recovery study. Psychon. Bull. Rev..

[82] White C.N., Curl R. (2017). A Spotlight Diffusion Model Analysis of the Attentional Networks Task. https://osf.io/h9b8v/.

[83] Huebner R., Steinhauser M., Lehle C. (2010). A dual-stage two-phase model of selective attention. Psychol. Rev..

[84] Huebner R., Tobel L. (2012). Does attentional selectivity in the flanker task improve discretely or gradually?. Front. Psychol..

[85] Grange J.A. (2016). Flankr: An R package implementing computational models of attentional selectivity. Behav. Res. Methods.

[86] Rogers R.D., Monsell S. (1995). Costs of a predictible switch between simple cognitive tasks. J. Exp. Psychol. Gen..

[87] Schmitz F., Voss A. (2012). Decomposing task-switching costs with the diffusion model. J. Exp. Psychol. Hum. Percept. Perform..

[88] Schmitz F., Voss A. (2014). Components of task switching: A closer look at task switching and cue switching. Acta Psychol..

[89] Miller J., Ulrich R. (2013). Mental chronometry and individual differences: Modeling reliabilities and correlations of reaction time means and effect sizes. Psychon. Bull. Rev..

[90] Lerche V., Voss A., Nagler M. (2017). How many trials are required for parameter estimation in diffusion modeling? A comparison of different optimization criteria. Behav. Res. Methods.

[91] Eid M. (2000). A multitrait-multimethod model with minimal assumptions. Psychometrika.

[92] Van Ravenzwaaij D., Donkin C., Vandekerckhove J. (2017). The EZ diffusion model provides a powerful test of simple empirical effects. Psychon. Bull. Rev..

[93] Akaike H., Petrov B., Csáki F. (1973). Information theory and an extension of the maximum likelihood principle. Proceedings of the 2nd International Symposium on Information Theory.

[94] Schwarz G. (1978). Estimating the Dimension of a Model. Ann. Stat..

[95] Schubert A.L., Hagemann D., Voss A., Bergmann K. (2017). Evaluating the model fit of diffusion models with the root mean square error of approximation. J. Math. Psychol..

[96] Clauset A., Shalizi C.R., Newman M.E.J. (2009). Power-Law Distributions in Empirical Data. SIAM Rev..

[97] Voss A., Nagler M., Lerche V. (2013). Diffusion Models in Experimental Psychology. Exp. Psychol..

[98] Jackson D.L., Gillaspy J.A.J., Purc-Stephenson R. (2009). Reporting practices in confirmatory factor analysis: An overview and some recommendations. Psychol. Methods.

[99] D’Agostino R.B., D’Agostino R.B., Stephens M.A. (1986). Graphical analyses. Goodness-of-Fit Techniques.

[100] Skrondal A., Laake P. (2001). Regression among factor scores. Psychometrika.

[101] Frischkorn G.T., Schubert A.L., Neubauer A.B., Hagemann D. (2016). The Worst Performance Rule as Moderation: New Methods for Worst Performance Analysis. J. Intell..

[102] Lee M.D. (2011). How cognitive modeling can benefit from hierarchical Bayesian models. J. Math. Psychol..

[103] Lee M.D., Wagenmakers E.J. (2013). Bayesian Cognitive Modeling: A Practical Course.

[104] Kruschke J.K., Liddell T.M. (2017). The Bayesian New Statistics: Hypothesis testing, estimation, meta-analysis, and power analysis from a Bayesian perspective. Psychon. Bull. Rev..

[105] Vandekerckhove J., Tuerlinckx F., Lee M.D. (2011). Hierarchical diffusion models for two-choice response times. Psychol. Methods.

[106] Hamaker E.L., Dolan C.V., Molenaar P.C.M. (2005). Statistical Modeling of the Individual: Rationale and Application of Multivariate Stationary Time Series Analysis. Multivar. Behav. Res..

[107] Heck D.W., Arnold N.R., Arnold D. (2017). TreeBUGS: An R Package for Hierarchical Multinomial-Processing-Tree Modeling. Behav. Res. Methods.

[108] Bürkner P.C. (2017). brms: An R Package for Bayesian Multilevel Models Using Stan. J. Stat. Softw..

[109] Nunez M.D., Gosai A., Vandekerckhove J., Srinivasan R. (2018). The latency of a visual evoked potential tracks the onset of decision making. bioRxiv.

[110] Kelly S.P., O’Connell R.G. (2013). Internal and External Influences on the Rate of Sensory Evidence Accumulation in the Human Brain. J. Neurosci..

[111] O’Connell R.G., Dockree P.M., Kelly S.P. (2012). A supramodal accumulation-to-bound signal that determines perceptual decisions in humans. Nat. Neurosci..

[112] Box G.E.P. (1976). Science and Statistics. J. Am. Stat. Assoc..

